# Mechanochemical Recycling of Flexible Polyurethane Foam Scraps for Quantitative Replacement of Polyol Using Wedge-Block-Reinforced Extruder

**DOI:** 10.3390/polym16121633

**Published:** 2024-06-09

**Authors:** Lei Guo, Fu Wang, Hailin Chai, Gongxu Liu, Xingao Jian, Jinyang Zhao, Kexin Liu, Haichao Liu, Tiewei Liu, Xiangping Zhang, Yongshuai Wang, Fumin Liu

**Affiliations:** 1 College of Electromechanical Engineering, Qingdao University of Science & Technology, Qingdao 266061, China; 06weny@163.com (L.G.); 2023032012@mails.qust.edu.cn (F.W.); 4022030005@mails.qust.edu.cn (H.C.); 4021030022@mails.qust.edu.cn (G.L.); 4022030024@mails.qust.edu.cn (X.J.); 4022030073@mails.qust.edu.cn (J.Z.); 4022030033@mails.qust.edu.cn (K.L.); 2 National Engineering Laboratory of Advanced Tire Equipment and Key Materials, Qingdao University of Science & Technology, Qingdao 266061, China; liuhaichao@qust.edu.cn; 3 Hisense Refrigerator Co., Ltd., Qingdao 266700, China; liutiewei@hisense.com (T.L.); zhangxiangping@hisense.com (X.Z.); wangyongshuai@hisense.com (Y.W.)

**Keywords:** mechanochemical recycling, flexible polyurethane foam, wedge-block-reinforced extruder, de-crosslinking, polyol quantitative replacement

## Abstract

Recycling flexible polyurethane foam (F-PUF) scraps is difficult due to the material’s high cross-linking structure. In this work, a wedge-block-reinforced extruder with a considerable enhanced shear extrusion and stretching area between the rotating screw and the stationary wedge blocks was utilized to recycle F-PUF scraps into powder containing surface-active hydroxyl groups. The powder was then utilized for the quantitative replacement of polyol in the foaming process. Characterizations showed that the continuous shear extrusion and stretching during the extrusion process reduced the volume mean diameter (VMD) of the F-PUF powder obtained by extruding it three times at room temperature to reach 54 μm. The -OH number (OHN) of the powder prepared by extruding it three times reached 19.51 mgKOH/g due to the mechanochemical effect of the powdering method. The F-PUF containing recycled powder used to quantitively replace 10 wt.% polyol was similar in microstructure and chemical structure to the original F-PUF, with a compression set of 2%, indentation load deflection of 21.3 lbf, resilience of 43.4%, air permeability of 815.7 L/m^2^·s, tensile strength of 73.0 Kpa, and tear strength of 2.3 N/cm, indicating that the recycling method has potential for industrial applications.

## 1. Introduction

Polyurethane (PU) materials rank sixth in terms of global polymer production, boasting an annual output ranging between 18 and 24 million tons [1,2,3]. Among the PU materials, polyurethane foam (PUF), which is made by reacting polyether polyol or polyester polyol with aromatic diisocyanate, constitutes approximately 70% of this impressive figure. PUF can be divided into two categories: flexible polyurethane foam (F-PUF) and rigid foam (R-PUF). F-PUF accounts for over 50% of the PUF market and has been widely used in fields such as construction, transportation, and furniture due to its light weight, softness, and durability. However, with the explosive growth of production, the treatment of F-PUF scraps and waste has become an important environmental issue [4,5,6]. Currently, landfilling remains the preferred method in many countries for the treatment of PUF waste due to its lower cost, accounting for approximately 30.8% of the total waste [7]. However, the slow natural decay of PU materials leads to serious environmental hazards in landfilling treatment. Another 39.5% of PUF waste is managed through energy recovery [7], but poisonous gas may be generated during combustion, which can lead to the greenhouse effect [8]. Following policies toward zero waste plastic and carbon neutrality, the recycling of PUF scraps and waste as reusable materials in the substitution of raw materials is an attractive alternative. However, since F-PUF is a kind of non-melting elastic thermosetting material with a high cross-linking structure, it is very difficult recycle [9,10].

Research on the recycling of PUF mainly focuses on chemical and mechanical recycling methods [11]. Chemical recycling methods aim to transform PUF scraps and waste into other useful compounds, mainly polyol, through the chemical depolymerization reaction. Based on the chemical reagents used in the chemical depolymerization reaction, chemical recycling methods can be divided into hydrolysis [12,13], glycolysis [14,15,16], acidolysis [17,18,19], and aminolysis [20,21,22]. However, due to the complexity of product purification and the high cost, only glycolysis and acidolysis have been tested at the pilot scale [23,24]. In addition, the toxic byproducts produced during the glycolysis and acidolysis processes means that these methods have not been commercialized [25]. By contrast, mechanical recycling methods are more economical and have fewer toxic byproducts [26]. Mechanical recycling methods aim to crush the PUF scraps and waste into small particles for further processing (such as injection molding or extrusion) [27], which can be classified into regrinding [28], rebonding [29], adhesive pressing [30], and molding [31]. However, as PU is a thermosetting material, it cannot be remolded during the mechanical recycling process. Therefore, the material recovered through mechanical recycling methods can only be used for the preparation of low-value products. Recently, another kind of recycling method, the mechanochemical method, has been receiving increasing attention [32]. The mechanochemical method aims to break the crosslinking bonds of PUF during the process of making scraps and waste into powder that can be reutilized as a partial substitute for polyol. Beran et al. [33] crushed R-PUF into powder using two-roll milling and reutilized the recycled powder as a polyol replacement in polyurethane adhesives. Duan et al. [34] also recycled R-PUF into partial de-crosslinking powder through the solid-state shear grinding method and prepared composites with plasma-treated modified regenerated polyester fibers as reinforcing fillers through compression molding. We also used a two-roll mill to grind F-PUF scraps into an active powder through strong shear regrinding and then utilized it as a partial substitute for polyol [35]. However, in all of the research mentioned above, there has been no quantitative evaluation of the reactivity of recycled PUF powder. Although the -OH number (OHN) was evaluated for the powder and polyol mixture in Beran’s work [33], the results cannot determine the -OH number of the recycled powder. This may be due to the large particle size of the recovered PUF powder, causing steric hindrance and reducing the reactivity of surface-active groups.

In this study, F-PUF scraps were ground into a powder with a smaller particle size and containing surface-active hydroxyl groups using strong shear extrusion and stretching using a single-screw extruder reinforced with a wedge block. The powder was then utilized as a quantitative replacement for polyol in the foaming process. The fine pulverization and mechanochemical effect mechanism of the recycled powder was analyzed and verified by a series of characterizations. The morphologies, Fourier-transform infrared spectroscopy (FTIR) results, and the macro properties of the F-PUF containing recycled powder and the original F-PUF were compared, and the influence of the recycled powder on the re-foaming products was evaluated.

## 2. Materials and Methods

### 2.1. Materials

F-PUF scraps: flexible low-density polyurethane foam 2420 (density of 24 kg/m^3^, indentation load deflection (ILD) > 20 lbf, compression set < 3%, Foshan Musi Furniture Co., Ltd., Foshan, China).

Raw materials used for F-PUF preparation (re-foaming): toluene diisocyanate (TDI) (T80, a product made by mixing 2,4-toluene diisocyanate and 2,6-toluene diisocyanate in a ratio of 80:20, Wanhua Chemical Group Co., Ltd., Yantai, China), polyether polyol (5616, viscosity of 980 mPa·s, molecular weight of 3000, functionality of 3, OHN of 56 mg KOH/g, water content ≤ 0.05%, Wanhua Chemical Group Co., Ltd., Yantai, China), polymer polyol (LHS-100, molecular weight of 3500, functionality of 2, OHN of 32 mg KOH/g, water content ≤ 0.08%, Shandong Longhua New Material Co., Ltd., Zibo, China), deionized water, triethylenediamine (A33, mixed with 80% 3156, Wanhua Chemical Group Co., Ltd., Yantai, China), dichloromethane (MC, Changhua Chemical Technology Co., Ltd., Suzhou, China), divalent tin (Dabco T-9, Guangzhou Innovate Chemical Co., Ltd., Guangzhou, China), and silicone oil (L618, mixed with 80% 3156, Wanhua Chemical Group Co., Ltd., Yantai, China).

### 2.2. Preparation of Samples

**Figure 1:**
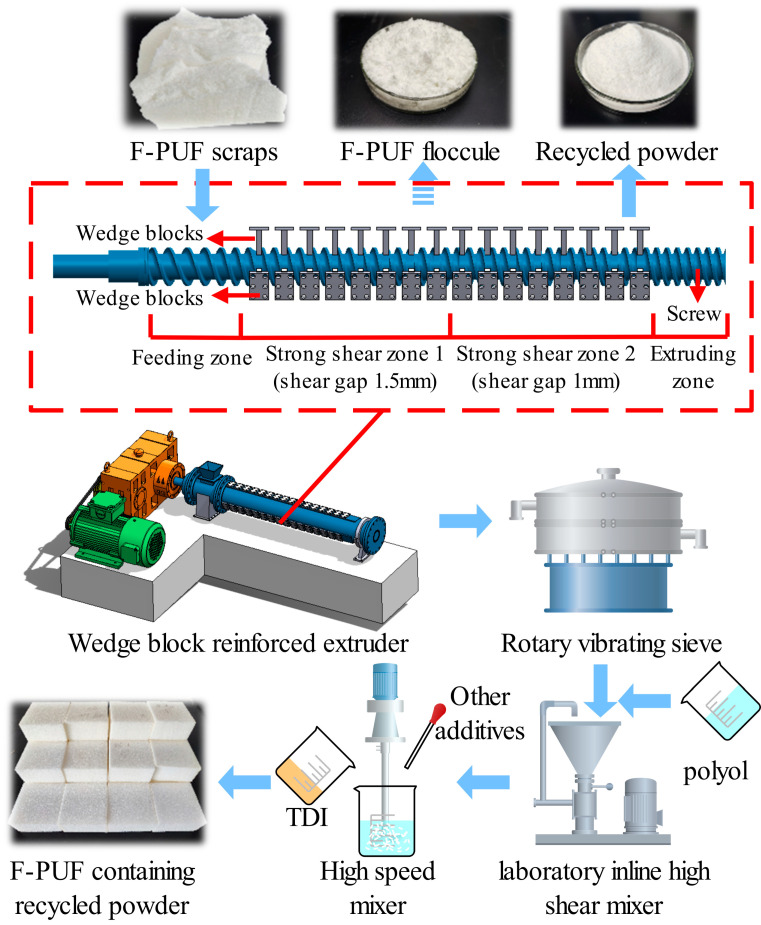
Schematic diagram of the recycling process.

The mechanochemical recycling process consists of three steps: the preparation of the recycled F-PUF powder, the mixing of polyether and the recycled powder, and the preparation of the F-PUF containing the recycled powder, as shown in Figure 1.

#### 2.2.1. Preparation of Recycled F-PUF Powder

Initially, the F-PUF scraps were roughly broken into strips approximately 15 cm wide and 4 cm thick for subsequent extruding. Then, the strips were transported via a feeding unit into the extruder for further crushing and extrusion with a screw speed of 30 r/min and a barrel temperature of 50 °C. Powder samples with different numbers of extrusion cycles (1, 3, and 5) were prepared. Finally, all the recycled powder output from the extruder head was sifted using an 80 mesh sieve.

#### 2.2.2. Mixing of Polyol and the Recycled Powder

The recycled powder was mixed with the polyether polyol using the laboratory’s BZ1-1.5 inline high shear mixer (Anhui Bunkin Chemical Machinery Co., Ltd., Hefei, China). The mass of each of the powder–polyol mixtures was 300 g. And the recycled powder–polyol mixtures made in this work had a powder content ranging from 5% to 30%. The mass of the recycled powder and polyether polyol used in each mixture varied depending on the powder content of the mixture. The mixer rotated 3000 times a minute, and the mixing time was 30 min.

#### 2.2.3. Preparation of F-PUF Containing Recycled Powder

Table 1 presents the formula of the original F-PUF. While preparing the F-PUF containing recycled powder, the polyol in the formula was replaced with the powder–polyol mixture, and the TDI mass fraction was adjusted based on the OHN of the powder–polyol mixture under the condition of an unchanged isocyanate index to quantitatively replace the polyol. The raw materials, except TDI, were stirred at 1000 r/min for 1 min using the high-speed SN-OES-200SH mixer (Shanghai Shangpu Instrument Equipment Co., Ltd., Shanghai, China). Subsequently, TDI 80 was added, and the whole mixture was stirred again for 8 s at 3000 r/min. The mixture was subsequently poured into a carton mold for foaming. Finally, the foam was left at room temperature for 24 h to mature.

## 3. Characterization

### 3.1. Characterization of Recycled Powder

The particle size distribution of the recycled powder was measured using the HELOS (H3839) laser granulometer and the SUCELL2, R5 (SYMPATEC GmbH, Clausthal-Zellerfeld, Germany). The powder samples were first stirred in deionized water. The size range obtained using this technique was from 0.1 to 875 μm. PAQXOS 3.0.2 software was utilized to calculate the volume mean diameter (VMD).

The temperature of the recycled powder after the extrusion process was measured by the 3i Smart LT infrared thermometer (Fluke Process Instruments (Shanghai) Co., Ltd., Shanghai, China).

The morphologies of the recycled powder were characterized by scanning electron microscopy (SEM) using the SU8000 (Hitachi High Tech Co., Ltd., Tokyo, Japan). The powder was first stirred evenly in deionized water, then dried on a cover glass, and finally, gold-plated before undergoing SEM imaging.

The chemical structure of the recycled powder was confirmed with the FTIR EQUINOX 55 (BRUKER Co., Karlsruhe, Germany) in the attenuated total reflection (ATR) mode with a scan range of 4000–500 cm^−1^.

The crosslinking density was evaluated using nuclear magnetic resonance (NMR) with the VTMR20-010V-I (Suzhou NUMAG Analytical Instrument Co., Ltd., Suzhou, China). The test temperature was 90 °C, and the ^1^H transverse relaxation curve was measured using the CPMG pulse sequence.

### 3.2. Characterization of Powder–Polyol Mixture

The viscosity of the powder–polyol mixture was measured with the NDJ-1 rotary viscometer (Shanghai Precision Scientific Instrument Co., Ltd., Shanghai, China) using spindle No. 3. All the samples were measured at 25 °C.

The OHN was determined according to the ASTM D4274-23 Test C standard. A 0.5 N NaOH solution was utilized for the titrations. The OHN was calculated according to Equation (1):

(1) $$\mathrm{OHN}=\left[(B-A)N\times 56.1\right]/W$$

where *A* is the volume of the NaOH solution used for titration (mL); *B* is the volume of the NaOH solution used for blank titration (mL); *N* is the normality of the NaOH solution; *W* is the sample weight (g).

### 3.3. Characterization of F-PUF (Including the F-PUF Containing Recycled Powder and the Original F-PUF)

The density of the F-PUF was determined according to the ASTM D3574-17 Test A standard, and the samples mass was measured using the JA3003C high-precision electronic balance (Shanghai Yueping Scientific Instrument Co., Ltd., Shanghai, China).

The resilience of the F-PUF was measured in accordance with the ASTM D3574-17 Test H standard using a ball rebound tester PMLQ-500 (Beijing Guance Jingdian Equipment Co., Ltd., Beijing, China).

The ILD was determined according to the ASTM D3574-17 Test B1 standard with a spongy indentation hardness testing machine PMYX-2000 (Beijing Guance Jingdian Equipment Co., Ltd., Beijing, China).

According to the ASTM D395-18 standard, the compression set of the F-PUF was measured using a compression set tester YSBX-1 (Beijing Guance Jingdian Equipment Co., Ltd., Beijing, China). The sample was compressed to 25% of its initial thickness for 22 h at 70 °C, and the thickness recovery after removing the strain was measured after the sample was taken out and left to stand for 30 min. The compression set value was calculated according to Equation (2):

(2) $$Cs=(t_{0}-t_{f})/t_{0}\times 100$$

where *t*_0_ and *t_f_* are the original thickness and final thickness, respectively.

The air permeability was determined according to ASTM D3574-17 Test G with the SG461-III sponge air permeability testing instrument (Changzhou Shuanggudunda Electromechanical Technology Co., Ltd., Changzhou, China).

The tensile strength was determined according to ASTM D3574-17 Test E with the UH4503 sponge tensile and tear strength testing machine (Shanghai Heng Yu Instrument Co., Ltd., Shanghai, China).

The tear strength was determined according to ASTM D3574-17 Test F with the UH4503 sponge tensile and tear strength testing machine (Shanghai Heng Yu Instrument Co., Ltd., Shanghai, China).

The morphologies of the F-PUF were observed using the same SEM used for powder characterization. The samples used to observe the cell structure and wall thickness were cryogenically fractured in liquid nitrogen to produce a cross-sectional surface.

The FTIR spectra were obtained using the same method as described in Section 3.1.

## 4. Results and Discussion

### 4.1. Effects of Room-Temperature Wedge-Block-Reinforced Extrusion Recycling Method on F-PUF

#### 4.1.1. Mechanism and Characterization of Fine Pulverization

Reducing the size of F-PUF at room temperature is challenging due to its elastic nature. It was reported that the minimum particle size of F-PUF powder was about 100 μm when crushed by a two-roll mill [36]. During the two-roll milling process, strong shear compression and stretching forces are generated in the narrow gap between two rollers, acting on the polyurethane chains to overcome the resilience to produce extremely high internal stress. The VMD of the powder decreases as the milling cycle increases initially [35]. However, the time for the powder to pass through the roller gap process is very short, resulting in a lack of sustained internal stress in the polyurethane chains, which limits further reduction of the particle size. In the wedge-block-reinforced extruder, the wedge blocks are evenly distributed radially along the screw in the strong shear zone (with an angle of 120° between adjacent wedge blocks) The gap height between the bottom (or side) of the wedge blocks and the bottom (or side) of the screw groove is 1.5 mm and 1 mm in the strong shear zones 1 and 2, respectively, to gradually enhance the shear compression and stretching effects. During the room-temperature wedge-block-reinforced extrusion process, the rotation of the screw generates continuous shear extrusion and stretching, and the wedge blocks further enhance the shear extrusion and stretching effect. This makes it possible to achieve finer powder production of F-PUF with smaller particle sizes. As shown in Figure 1, the F-PUF scraps were broken into floccule after passing through the strong shear zone 1 in the first extrusion process, and then broken into powder after the first complete extrusion process.

**Figure 2:**
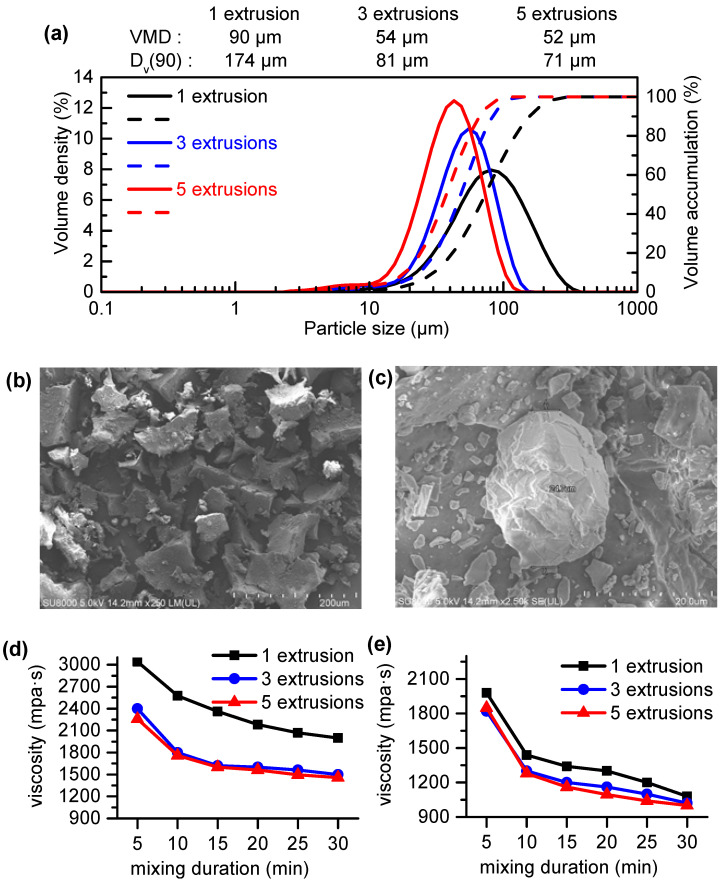
(**a**) Particle size distribution of the recycled F-PUF powder obtained using the room-temperature wedge-block-reinforced extrusion recycling method with varying numbers of extrusion cycles. (**b**) SEM photographs of the recycled F-PUF powder (magnification ×250). (**c**) SEM photographs of the recycled F-PUF powder (magnification ×2500). (**d**) Effects of mixing duration on the viscosity of the powder–polyol mixtures with the powder obtained under different numbers of extrusion cycles (powder content of 15 wt.%). (**e**) Effects of the mixing duration on the viscosity of the powder–polyol mixtures with the powder obtained under different numbers of extrusion cycles (powder content of 10 wt.%).

Figure 2a presents the particle size distribution of the recycled powder prepared using the room-temperature wedge-block-reinforced extrusion recycling method, with varying numbers of extrusion cycles. The solid line in the figure represents the volume density, while the dashed line represents the volume accumulation. It can be observed that with the increase in extrusion cycles, the powder particle size gradually decreased, and the particle size distribution tended to become concentrated. Compared to the powder prepared by one cycle of extrusion, the VMD and D_V_ (90) (the particle diameter corresponding to 90% of the cumulative particle size distribution) of the powder prepared by three cycles of extrusion were reduced by 40% and 53%, respectively. However, further increasing the number of extrusion cycles did not lead to a significant decrease in the particle size. Nonetheless, the VMD of the powder prepared by three extrusion cycles was 52 μm, which is approximately 50% of the minimum particle size reported in the current literature [36], verifying the mechanism of fine pulverization.

To further reveal the microstructure of the powder, microscopic morphologies are illustrated in Figure 2b,c. Due to the similarity in the physical structures of the powders prepared with different numbers of extrusion cycles, the microscopic morphologies of the powder prepared by three extrusion cycles and magnified by 250 times and 2500 times are provided as examples for analysis. As shown in Figure 2b, small particles were adsorbed on the surface of larger irregular particles. No pore structure is observed, while some dense and smooth cell walls are preserved. There is a multi-layer structure on the particle surface in Figure 2c, which is obviously the cross section caused by extrusion and stretching. This further illustrates the role of the enhanced extrusion stretching effect generated by the wedge blocks in reducing particle size during the room-temperature wedge-block-reinforced extrusion process.

It was reported that the viscosity of powder–polyol mixtures is significantly affected by the powder particle size [37]. To clarify whether the viscosities of the mixtures were within the allowable range, Figure 2d,e show the effects of the mixing duration on the viscosities of the powder–polyol mixtures with the powder obtained under different extrusion cycles and different powder contents. It should be mentioned that the polyol adopted in the experiment was obtained by mixing 5616 and LHS-100 at a proportion of 1:1 according to the preparation formula presented in Table 1. The initial viscosity of the mixed polyol was 980 mPa·s. It can be seen that for all powder–polyol mixtures, the viscosity significantly decreased in the initial 5 min of mixing. Although the viscosity decreased with an increase in the mixing duration, the decrease rate gradually slowed down. Figure 2d,e show that under the same mixing duration, the smaller the particle size of the powder, the lower the viscosity of the powder–polyol mixture. Comparing Figure 2d,e, it can be seen that an increase in the powder content resulted in an increase in the viscosity. Specifically, the maximum viscosity of the powder–polyol mixture containing 10 wt.% powder after mixing for 30 min was 1080 mPa·s, which is similar to the initial viscosity of the mixed polyol. This indicates that there was no significant increase in the viscosity of the powder–polyol mixture containing 10 wt.% powder prepared by the recycling method under a sufficient mixing duration. Based on the viscosity measurements, the powder content in the powder–polyol mixture used in the foaming process was set at 10 wt.%.

#### 4.1.2. Mechanism and Characterization of Mechanochemical Effect

**Figure 3:**
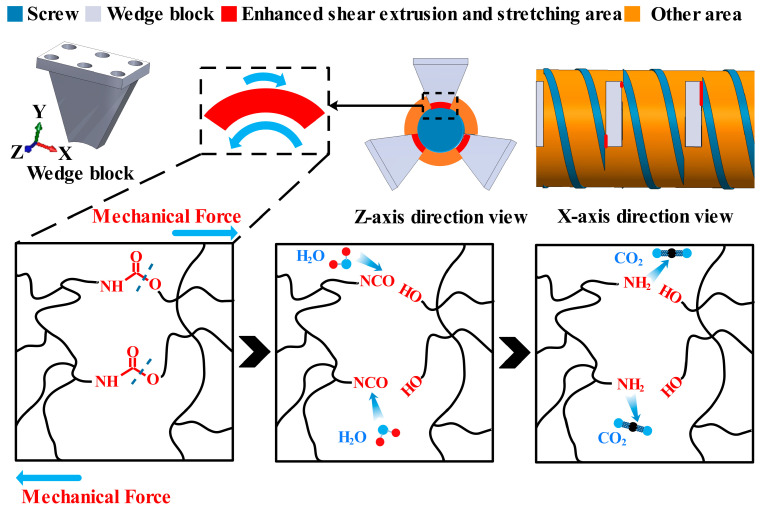
Schematic diagram of the mechanochemical effect.

The mechanochemical effect was explained in detail in our previous research [35]. The core mechanism is the high internal stress generated within the polyurethane segment by shear extrusion and stretching during the powdering process, which leads to both physical and chemical de-crosslinking. Physical de-crosslinking is caused by hydrogen bond breakage, while chemical de-crosslinking is the breakage of chemical bonds with lower stored energy due to stress deformation [38] in the polyurethane framework, most likely occurring at the C-O bonds in carbamate groups [39]. In the wedge-block-reinforced extruder, wedge blocks penetrate into the bottom of the screw groove, creating an extensive enhanced shear extrusion and stretching area between the rotating screw and the stationary wedge blocks. This includes the area between the bottom of the wedge blocks and the bottom of the screw groove (the red area shown in the *Z*-axis direction view in Figure 3) and the area between the side of the wedge block and the side of the screw edge (the red area shown in the *X*-axis direction view in Figure 3). The energy provided by these enhanced shear extrusion and stretching areas is higher than the C-O bond energy, causing the de-crosslinking of the C-O bonds in carbamate groups and forming isocyanate and hydroxyl groups, as illustrated in Figure 3. Among these, the isocyanate (-NCO) groups generated in one decomposition process are highly reactive and can immediately react with moisture in the air to form CO_2_ gas and amine groups [40]. Thus, the generated -OH groups are retained, making the recycled powder capable of replacing some polyol.

**Figure 4:**
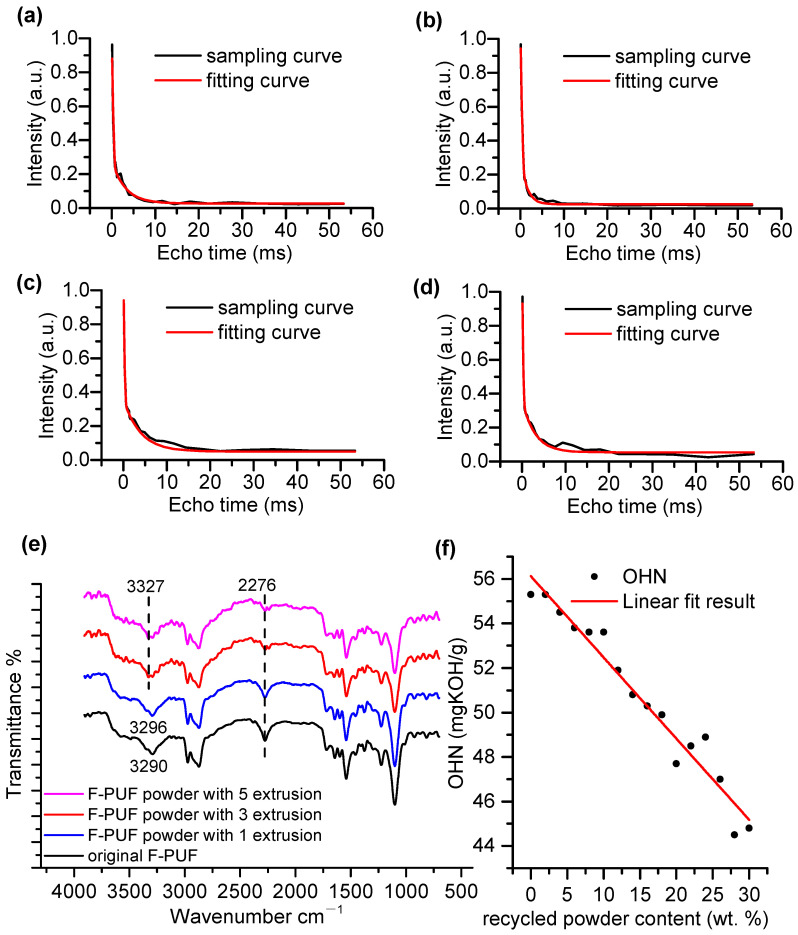
(**a**) The ^1^H lateral relaxation curve and its fitting curves of the original F-PUF. (**b**) The ^1^H lateral relaxation curve and its fitting curve of the F-PUF powder with 1 extrusion. (**c**) The ^1^H lateral relaxation curve and its fitting curve of the F-PUF powder with 3 extrusions. (**d**) The ^1^H lateral relaxation curve and its fitting curve of the F-PUF powder with 5 extrusions. (**e**) The ATR-FTIR spectra of the recycled F-PUF powder and the original F-PUF. (**f**) The OHN of the powder–5616 polyol mixture with the powder content ranging from 5% to 30%, and the linear fit result.

To evaluate the mechanochemical de-crosslinking effect of the room-temperature wedge-block-reinforced extrusion recycling method, ^1^H lateral relaxation curves and their fitting curves for the original F-PUF and the recycled powder obtained by different numbers of extrusion cycles are presented in Figure 4a–d. The fitting curves were obtained using XLD, which can be expressed as Equation (3):

(3) $$\frac{M(t)}{M(0)}=A_{1}\exp(-\frac{t}{T_{2}}-0.5qM^{rl}t^{2})+B_{1}\exp(-\frac{t}{T_{2}})+A_{0}$$

where *M*(*t*)/*M*(0) is the normalized lateral relaxation value, *M*(*t*) is the lateral relaxation value at time *t*; *M*(0) is the lateral relaxation value at time *t* = 0; *A*_1_ and *B*_1_ represent the content of cross-linking and dangling chains, respectively; *T*_2_ is the average ^1^H lateral relaxation time of the cross-linked and suspended segments; *q* is the anisotropic parameter; *M^rl^* is the dipole moment in rigid lattice molecules; *A*_0_ is a DC component without actual physical significance.

It can be observed that the lateral relaxation curve of the recycled powder is similar to that of the original F-PUF. This similarity is due to the de-crosslinking caused by the mechanochemical effects predominantly occurring on the surface of the recycled F-PUF powder, while the cross-linking structure inside the powder remained basically unchanged. The fitting parameters *A*_1_ and *B*_1_ are presented in Table 2 to describe the subtle difference in the crosslinking density between the original F-PUF and the recycled powder. To demonstrate the statistical significance of the data, three independent tests were conducted on each sample, and the averages and standard deviations of the fitting parameters were also provided. Compared to the original F-PUF, the cross-linking proportion of the chains in the powder prepared by the three processes was reduced. When the number of extrusion cycles is small, the cross-linking proportion decreased with increasing extrusion cycles, which indicates that the OHN of the foam increased with the increase in the number of extrusion cycles according to the mechanism of the mechanochemical effect. However, when the number of extrusion cycles exceeded three, further increasing the number of extrusion cycles had no significant effect. About 4% of the chains changed from cross-linking chains to dangling chains after three extrusion cycles, verifying the mechanochemical de-crosslinking effect.

To clarify the location of chain breakage and the de-crosslinking products, the ATR-FTIR spectra of the original F-PUF and the recycled powder obtained by different numbers of extrusion cycles are utilized, as shown in Figure 4e. The absorption peaks at 2276 and 3290 cm^−1^ were assigned to the stretching vibrations of the -NCO groups and -NH groups, respectively. It can be seen that the absorption peak of the -NH groups increased after the room-temperature wedge-block-reinforced extrusion process, affected by the -OH groups generated in the chemical de-crosslinking. The residual -NCO groups in the original F-PUF, as well as those generated in the chemical de-crosslinking, reacted with moisture to generate amine groups and CO_2_ gas during the powdering process, resulting in a decrease in the peak value at 2276 cm^−1^. As a result, the -OH groups generated during the powdering process were retained. Compared to the powder prepared by one extrusion cycle, the absorption peak of -NH groups increased and the peak strength of -NCO groups decreased for the powder prepared by three extrusion cycles. However, the ATR-FTIR spectrum of the powder prepared by three extrusion cycles is similar to that of the powder prepared by five extrusion cycles. This is consistent with the crosslinking density measurement results, indicating that utilizing the room-temperature wedge-block-reinforced extrusion recycling method with three extrusion cycles is relatively efficient and economical. Thus, the powder used in the OHN measurement and foaming process was prepared by three extrusion cycles. It should be noted that the powder temperature at the outlet of the extruder barrel was about 45 °C, which means that the -OH groups were not generated by the thermal decomposition of the polyurethane but by the mechanochemical reactions.

To further quantitatively clarify the ability of the recycled powder prepared by three extrusion cycles to replace polyol, the OHN of the powder–5616 polyol mixture with a powder content ranging from 5% to 30% and the linear fit result are given in Figure 4f. The fitting equation is shown in Equation (4):

(4) y=56.12−0.3649x

where *y* is the OHN, and *x* is the powder content. The adjusted R-square is 0.9526, indicating a high degree of fit between the equation and the measured values. The fitting result shows that for each phr recycled powder added to replace polyol, the OHN decreased by 0.3649. In theory, for every phr polyether polyol reduced, the OHN decreases by 0.56. This indicates that the OHN of the powder is 19.51 mgKOH/g, approximately 35% of the polyol. It should be noted that the water content of the powder–5616 polyol mixture containing 30 wt.% powder was only 0.08%, which is close to the water content of 5616 polyol, indicating that the change in the OHN was not caused by the adsorbed humidity on the powder surface.

### 4.2. Properties of F-PUF Products

**Figure 5:**
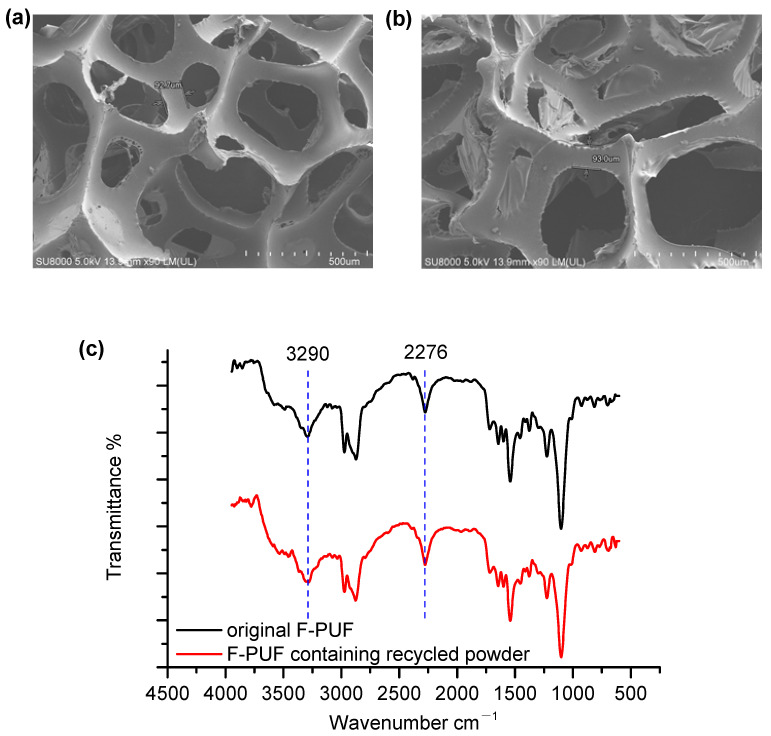
(**a**) Microscopic morphologies of F-PUF containing recycled powder (10 wt.% in the powder–polyol mixture). (**b**) Microscopic morphologies of original F-PUF. (**c**) ATR-FTIR spectra of the F-PUF containing powder prepared and the original F-PUF.

The microscopic morphologies of the F-PUF containing recycled powder prepared by three extrusion cycles (10 wt.% in the powder–polyol mixture) and the original F-PUF at a magnification of ×90 are exhibited in Figure 5a,b to evaluate the impact of recycled powder introduction on the cell structure. It can be seen that the pore structure of the F-PUF containing recycled powder is similar to that of the original F-PUF, and the cell edge thickness of both samples is about 90 μm, which is only slightly larger than the Dv (90) of the recycled powder. Nevertheless, there is no discernible recycled powder embedded on the cell edge in the F-PUF containing recycled powder. Therefore, the recycled powder had no obvious agglomeration in the foam and may have mainly existed at the intersections of multiple cell edges, indicating that the recycled powder plays the role of cross-linking points because of the -OH groups on its surface.

To elucidate the changes in the chemical structure of the F-PUF after replacing the polyols with the recycled powder, the ATR-FTIR spectra of the F-PUF containing powder prepared by three extrusion cycles and the original F-PUF are compared in Figure 5c. Although the absorption peaks of the recycled F-PUF powder and the original F-PUF are different at 2276 and 3290 cm^−1^, the ATR-FTIR spectra of the PUF containing recycled powder are basically the same as the original F-PUF. This indicates that by measuring the hydroxyl value of the powder and quantitatively replacing the polyol, the isocyanate index of the F-PUF products remained unchanged and the chemical structure was stable.

To evaluate the macroscopic properties of the F-PUF containing powder, measurements of its density, resilience, ILD, compression set, air permeability, tensile strength and tear strength were conducted and compared with those of the original F-PUF, as indicated in Table 3. It can be seen that there was almost no difference in the compression set, resilience, tensile strength, or tear strength between the two samples. This proves that there was no obvious stress concentration on the interface between the recycled powder and the original system during the compression process, which could have led to the destruction of the cell structure. However, the density of the F-PUF containing recycled powder decreased. This may be because the reactivity of -OH groups on the recycled powder surface decreased due to steric hindrance, leading to a rate of polymerization less than the bubbling rate during the foaming process. The decrease in density also caused a decrease in the ILD and an increase in air permeability. Nevertheless, the F-PUF containing recycled powder still met the ILD requirements of the original foam, indicating that the mechanochemical recycling method has market application value.

## 5. Conclusions

In this study, a room-temperature wedge-block-reinforced extrusion recycling method was proposed to recycle F-PUF scraps into powder containing surface active hydroxyl groups. In the room-temperature wedge-block-reinforced extrusion process, the rotation of the screw generated continuous shear extrusion and stretching to prepare small-particle-sized F-PUF powders, and extensive enhanced shear extrusion and stretching areas were created between the rotating screw and the stationary wedge blocks to enhance the mechanochemical effect. This caused the de-crosslinking of the C-O bonds in carbamate groups and formed isocyanate and hydroxyl groups. The VMD and D_V_ (90) of the recycled powder obtained by three extruding cycles were 54 μm and 81 μm, respectively. About 4% of the chains changed from cross-linking chains to dangling chains after three extrusion cycles, and the OHN of the recycled powder was 19.51 mgKOH/g. The powder temperature at the outlet of the extruder barrel was about 45 °C, verifying that the -OH groups were not generated by thermal decomposition of the polyurethane but by the mechanochemical reactions.

Due to the reactive activity of the recycled powder, it was utilized by quantitatively replacing 10 wt.% of the polyol during the foaming process. The viscosity of the powder–polyol mixture containing 10 wt.% powder after mixing for 30 min was similar to the initial viscosity of the mixed polyol. The F-PUF containing recycled powder to quantitatively replace 10 wt.% polyol was similar in microstructure and chemical structure to the original F-PUF, with resilience of 43.4%, ILD of 21.3 lbf, air permeability of 815.7 L/m^2^·s, compression set of 2%, tensile strength of 73.0 Kpa, and tear strength of 2.3 N/cm, indicating that the room-temperature wedge-block-reinforced extrusion recycling method has enormous potential in high-value recycling of F-PUF.

## Figures and Tables

**Table 1:** Formulas of the original F-PUF and F-PUF containing recycled powder to replace 10 wt.% polyol.

Sample	Component Mass (g)								
	Recycled Powder	5616	LHS-100	T-80	A33	T9	5870	MC	Deionized Water
Original F-PUF	0	150	150	133.05	0.66	0.54	3.6	12	9.9
F-PUF containing recycled powder	15	135	150	132.08	0.66	0.54	3.6	12	9.9

**Table 2:** Cross-linking density measurement by NMR.

Sample	Cross-Linking Chains Proportion A1 (%)	Dangling Chains Proportion B1 (%)
Original F-PUF	72.73 ± 0.14	27.27 ± 0.14
F-PUF powder with 1 extrusion	70.30 ± 0.29	29.70 ± 0.29
F-PUF powder with 3 extrusions	68.90 ± 0.28	31.10 ± 0.28
F-PUF powder with 5 extrusions	68.43 ± 0.43	31.25 ± 0.43

**Table 3:** Macroscopic properties of F-PUF product.

Sample	Density (Kg/m^3^)	Resilience (%)	ILD (lbf)	Compression Set (%)	Air Permeability (L/m^2^·s)	Tensile Strength (Kpa)	Tear Strength (N/cm)
Original F-PUF	23.90 ± 0.21	43.6 ± 0.4	26.4 ± 0.3	2.0 ± 0.2	660.8 ± 0.6	73.1 ± 0.4	2.2 ± 0.2
F-PUF containing recycled powder	22.71 ± 0.18	43.4 ± 0.3	21.3 ± 0.2	2.0 ± 0.3	815.7 ± 0.7	73.0 ± 0.5	2.3 ± 0.2

## Data Availability

The data are contained within the article.
